# From neuroleptics to neuroscience and from Pavlov to psychotherapy: more than just the “emperor's new treatments” for mental illnesses?

**DOI:** 10.15252/emmm.201606650

**Published:** 2016-09-12

**Authors:** Jürgen Margraf, Silvia Schneider

**Affiliations:** ^1^ Mental Health Research and Treatment CenterDepartment of Clinical Psychology and PsychotherapyUniversity of BochumBochumGermany

**Keywords:** Neuroscience

## Abstract

After decades of proclaimed therapeutic breakthroughs, neither neurobiology nor neuroscience has led to better long‐term outcomes for any of the major mental disorders. This contrasts with the long‐term efficacy of psychosocial interventions and points to the necessity to focus on sustained success, broaden our concept of mental health problems, and resist the temptations of marketing.

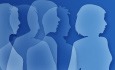

“The drugs don't work” was one of the hit singles from The Verve's album *Urban Hymns*, released in 1997. The song was written by lead singer Richard Ashcroft relating to his drug abuse, but it might well relate to the modern treatments for mental illnesses. More than half a century after neuroleptics, antidepressants, benzodiazepines, antipsychotics, behavior therapy, and cognitive treatment were introduced, it is prudent to ask whether “the drugs don't work”.

During the past 50 years, the industrialized world has seen a dichotomy between loudly proclaimed therapeutic breakthroughs and rapidly rising numbers of people on disability payments because of mental illness. We hear that antipsychotic, antidepressant, and anti‐anxiety drugs as well as behavior therapy and newer treatments have radically improved what was described as a dismal fate of people suffering from mental disorders. Simultaneously, the percentage of disabled mentally ill patients in the USA has risen by more than 600% since the 1950s (Whitaker, 2010) and similar rates are seen in European countries. Most epidemiologists agree that this “epidemic” is not caused by increased incidence. Moreover, the once rapid succession of new therapeutic developments seems to have halted, at least in pharmacology, as big companies are withdrawing from research on mental disorders.

How can this apparent contradiction be explained? Could it be that therapeutic progress is much less than we think or are being told? Could it be that the course of depression, anxiety, schizophrenia, or ADHD has been altered for the worse? Could it be that we cannot make therapeutic progress because the concept of mental illness and its treatment is deeply flawed? There are strong reasons to assume that all three suspicions are in fact true.

So, what do we know about the efficiency of pharmacological and psychological treatments? In regard to short‐term outcomes, pharmacotherapy is clearly inferior to cognitive behavior therapy (CBT) in treating anxiety disorders; for depression, the two modalities appear to be roughly equivalent, and most clinicians would argue that drug treatments are superior to psychotherapy for treating psychotic disorders. Neither drugs nor CBT show convincing efficacy against ADHD.

However, mental disorders are fluctuating and chronic conditions. What really counts therefore is lasting improvement. Here, the picture looks radically different: Lasting success after the end of treatment has only been shown for psychotherapy (typically CBT), whereas the effects of drug treatments vanish rapidly once the drugs are withdrawn. This is obvious for anxiety disorders, depression, and ADHD and may also apply to schizophrenia.

There are now plenty of data and evidence that, in the long term, the drugs do not work.

CBT undoubtedly outperforms drug treatments (benzodiazepines, antidepressants) for anxiety disorders such as panic and phobias. Since the 1980s, Western governments have also been warning that benzodiazepines are addictive and should not be used on a long‐term basis. Moderate to strong withdrawal syndromes, worsened anxiety, cognitive impairment, and functional decline are consistent consequences of long‐term use, and there is a clear dose–response relationship. Regarding depression, it was initially claimed that 70% of the patients responded to antidepressants and 30% to placebo. Today, these numbers are actually closer to 40 and 30% (Khan & Brown, 2015). The average effect size of antidepressants in trials submitted to the FDA is 0.30 (Gibertini *et al*, 2012), and when looking at clinical significance and “real‐world” patients, they are on average not better than placebo (Kirsch, 2010). Even more dubious results in children and adolescents led official institutions such as the UK's MHRA to conclude that most selective serotonin reuptake inhibitors (SSRIs) to treat depression are both ineffective and harmful.

With respect to long‐term results after end of treatment, psychotherapy, especially CBT, generally outperforms antidepressants (Voderholzer & Barton, 2016): For patients with major depression, relapse after withdrawal is the rule for antidepressants (50–80%, average around 60%) but the exception for psychotherapy (20–50%, on average around 30%). For anxiety disorders, the differences are even more pronounced: The lines for the effects of placebo and of active drugs cross shortly after treatment. In contrast, positive effects remain stable for most patients who had CBT and some even experience further gains. Similarly, it is now clear that stimulants show no long‐term efficacy against ADHD. Generally, adding psychotherapy to antidepressants yields better long‐term results, but adding antidepressants to psychotherapy does not improve the outcome of treatment. In fact, the combination of exposure—the hallmark CBT treatment—and benzodiazepines or tricyclics works less well than exposure alone for treating anxiety.

So perhaps, we should not withdraw medications at all? This is exactly what has happened during the past decade in the developed countries, and it has had a host of negative long‐term effects. Psychotropic medications are classically given for long periods of time, in reality often much longer than officially acknowledged. It is easily understandable that taking psychoactive drugs would alter the targeted neurotransmitter systems over time. Among negative clinical outcomes of long‐term antidepressant use are increasing chronicity and heightened relapse rates for depression and an elevated risk of moving from unipolar to bipolar affective disorder, especially in younger patients (Fava, 2003; Whitaker, 2010; Andrews *et al*, 2012). The continued use of antidepressants may also propel depression to a more malignant and treatment‐unresponsive course. Compensatory neural adaptations after drug withdrawal may result in “withdrawal symptoms and increased vulnerability to relapse” (Fava, 2003). In adolescents, use of stimulants and antidepressants may lead to juvenile bipolar illness. The negative effects of benzodiazepines need not be discussed again here.

What about neuroleptics to treat schizophrenia? Remarkably, seminal WHO studies established that the long‐term outcome of schizophrenia is consistently much better in “developing countries”, where only 15.9% of patients were continuously maintained on neuroleptics, than in “developed countries”, where 61% of patients received this treatment. Moreover, the outcomes for patients in the USA have gotten worse since the 1970s and were no better in 1994 than they had been in 1900 (Hegerty *et al*, 1994). Potential biological explanations for these disappointing results include drug‐induced dopaminergic supersensitivity, shrinkage of the frontal lobes, enlargement of the basal ganglia and a progressive loss of frontal white matter volume associated with a worsening of negative symptoms, increased functional impairment, and cognitive decline. Long‐term use of older neuroleptics, atypical antipsychotics, and clozapine is associated with smaller brain tissue volumes (white and gray matter) that cannot be attributed to severity of illness or substance abuse. In addition, there is the well‐known risk of permanently dysfunctional dopaminergic pathways, which result in tardive dyskinesia, tardive psychosis, and tardive dementia.

Most worrisome, however, are the effects in the developing brain of children and adolescents. Most mental disorders begin before the age of 14 and continue to evolve over the whole life span. Brain development persists into the early 20s and coincides with a main risk period for mental disorders and for negative effects of interfering with neurobiology. Basic animal research shows for instance that antipsychotic treatment during the childhood/adolescent period has long‐term effects on depressive‐like, anxiety‐like, and locomotor behaviors in adult rats (De Santis *et al*, 2016). Many other findings in animals support similar risks, and there are emerging data in humans, for instance, with respect to heightened suicide risk after SSRI treatments.

Given the enormous investments into research for the past 60 years, why are we not more successful in treating mental disorders? One reason may be the ill‐advised biological notion of mental illnesses. First, the “myth of chemical imbalance” (Kirsch, 2010). Based on the effects of drugs on various neurotransmitter systems, it was assumed that mental disorders result from deficiencies in these systems. This has now become a standard narrative to “explain” mental disorders to patients or the public at large. In order to qualify as a causal factor, however, the assumed pathophysiology would have to exist before the onset of the mental disorder. In contrast to various psychosocial risk factors, this has not been shown convincingly. Concerning the monoamine deficiency theory, for instance, “the incongruence between the scientific literature and the claims made in FDA‐regulated SSRI advertisements is remarkable, and possibly unparalleled” (Lacasse & Leo, 2005).

Second, the reification of diagnostic constructs (“depression”) as distinct illness categories leads to an uncritical acceptance of supposed qualitative differences between “health” and “illness”, whereas research shows equivocal or even outright contradictory results. Categories with dubious validity miss the relevant dimensions of human behavior to the great detriment of scientists, clinicians, and patients. One prominent alternative approach, the US National Institute's of Mental Health′s Research Domain Criteria, aims to develop a classification based on behavioral dimensions and neurobiological measures. Its units of analysis, however, range only from genes to self‐reports, curiously omitting the social level.

Third, although we have broadened our view since George Engel introduced the bio‐psycho‐social model of illness in 1977, we have interpreted this with an increasingly narrow focus on biology and “bottom‐up” causal pathways, largely neglecting “top‐down” causal pathways. This is in stark contrast to the clearly established relationships of mental health problems with psychosocial factors. Recent findings show that the effect of social factors is largely mediated by psychological mechanisms—sense of control, mental activity, delay of gratification, self‐efficacy, and so on. Moreover, there are now reliable findings that improving social factors also improves mental health in a lasting way.

After decades of proclaimed therapeutic breakthroughs and promises of imminent better treatments based on translation of basic science into clinical practice, neither neurobiology nor neuroscience has led to measurably better long‐term outcomes for any of the major mental disorders (Margraf & Zlomuzica, 2015). Although psychotropic drugs are by far the most often used treatment modality in industrialized countries, there is no compelling evidence for the long‐term stability of their small to moderate short‐term results. The scant follow‐up evidence points to high relapse rates once medication is withdrawn and substantial negative outcomes if it is not withdrawn.

What needs to be done? First, we need better collaboration in the right places: We need to tighten the interlocking of etiological and therapeutic research strategies and of the bio‐, psycho‐, and social levels of analysis of mental disorders. Second, we need a broad *and* a narrow focus. While we should not give up investigating clearly defined and measured biological processes, we need to complement this by a “broader” focus on psychological and social processes. Third, we need to push back on marketing. The marketing power of Big Pharma and parts of the life sciences and medicine enjoyed remarkable economic success with a more than fivefold increase in sales of psychotropic drugs in the USA since the 1980s. Today, almost a quarter of American women in their 40s and 50s regularly take antidepressants. Lastly, we need to focus on the doable. Rather than chasing chimeras—“magic bullet” pills or fashionable new psychotherapies—we need to make sure that treatments with proven long‐term efficacy reach those who need them.

A great example for a pragmatic approach that has achieved measurable success for millions of patients is the UK′s IAPT (Improving Access to Psychological Therapies) program, which provides evidence‐based short‐term psychotherapy for anxiety and depression. Indeed, the major problem in psychotherapy, in contrast to drug treatment, is not efficiency but availability for those in need and the quality of treatment. The cost is not a major issue: A standard CBT to treat anxiety disorders, for instance, costs less per year than a drug regimen.

A realistic assessment of our current treatment options and the close cooperation of clinicians and neuroscientists would help us to overcome the current stagnation and put us back on the track forward.

See Appendix for a further reading list.

## Supporting information

Appendix
