# Supplementary material for: From neuroleptics to neuroscience and from Pavlov to psychotherapy: more than just the “emperor's new treatments” for mental illnesses?
Source: EMBO Mol Med. 2016 Sep 12;8(10):1115–7. doi: 10.15252/emmm.201606650 (PMC5048361; doi:10.15252/emmm.201606650)
Supplement: Supplementary file 1 — Appendix [file EMMM-8-1115-s001.pdf]

Appendix: Further reading list

ADHD and methylphenidate:

Bolanos CA, Barrot M, Berton O, Wallace-Black D, Nestler EJ (2003) Methylphenidate treatment during pre- and periadolescence alters behavioral responses to emotional stimuli at adulthood. *Biol Psychiatry* 54: 1317–1329

Epstein JN, Casey BJ, Tonev ST, Davidson MC, Reiss AL, Garrett A, Hinshaw SP, Greenhill LL, Glover G, Shafritz KM *et al* (2007) ADHD- and medication-related brain activation effects in concordantly affected parent – child dyads with ADHD. *J Child Psychol Psychiat* 48: 899–913

Grund T, Lehmann K, Bock N, Rothenberger A, Teuchert-Noodt G (2006) Influence of methylphenidate on brain development – an update of recent animal experiments. *Behav Brain Funct* 2: 2

Le Noury J, Nardo JM, Healy D, Jureidini J, Raven M, Tufanaru C, Abi-Jaoude E (2015) Restoring Study 329: efficacy and harms of paroxetine and imipramine in treatment of major depression in adolescence. *BMJ* 351: h4320

Molina BS, Hinshaw SP, Swanson JM, Arnold LE, Vitiello B, Jensen PS, Epstein JN, Hoza B, Hechtman L, Abikoff HB *et al* (2009) The MTA at 8 years: prospective follow-up of children treated for combined-type ADHD in a multisite study. *J Am Acad Child Adolesc Psychiat* 48: 484–500

Sonuga-Barke EJ, Brandeis D, Cortese S, Daley D, Ferrin M, Holtmann M, Stevenson J, Danckaerts M, van der Oord S, Döpfner M *et al* (2013) Nonpharmacological interventions for ADHD: systematic review and meta-analyses of randomized controlled trials of dietary and psychological treatments. *Am J Psychiatry* 170: 275–289

Storebø OJ, Krogh HB, Ramstad E, Moreira-Maia CR, Holmskov M, Skoog M, Nilausen TD, Magnusson FL, Zwi M, Gillies D *et al* (2015) Methylphenidate for attention-deficit/hyperactivity disorder in children and adolescents: Cochrane systematic review with meta-analyses and trial sequential analyses of randomised clinical trials. *BMJ* 351: h5203

Affective disorders and antidepressants:

Angermann CE, Gelbrich G, Störk S, Gunold H, Edelmann F, Wachter R, Schunkert H, Graf T, Kindermann I, Haass M *et al* (2016) Effect of escitalopram on all-cause mortality and hospitalization in patients with heart failure and depression: the MOOD-HF randomized clinical trial. *JAMA* 315: 2683-2693

Ebmeier KP, Donaghey C, Steele JD (2006) Recent developments and current controversies in depression. *Lancet* 367: 153–167

Fava GA (2002) Long-term treatment with antidepressant drugs: the spectacular achievements of propaganda. *Psychother Psychosom* 71: 127-132

Fava GA, Offidani E (2011) The mechanisms of tolerance in antidepressant action.

Prog Neuropsychopharmacol Biol Psychiatry 35: 1593-1602

Moret C, Isaac M (2009) Problems associated with long-term treatment with selective serotonin reuptake inhibitors. *J Psychopharmacol* 23: 967-974

Sharma V, Sommerdyk C (2013) Are antidepressants effective in the treatment of postpartum depression? A systematic review. *Prim Care Companion CNS Disord* 15: PCC.13r01529

Viguera AC, Baldessarini RJ, Friedberg J (1998) Discontinuing antidepressant treatment in major depression. *Harv Rev Psychiat* 5: 293–306

Anxiety disorders and benzodiazepines:

Barker MJ, Greenwood KM, Jackson M, Crowe SF (2004) Cognitive effects of long-term benzodiazepine use: a meta-analysis. *CNS Drugs* 18: 37-48

Barker MJ, Greenwood KM, Jackson M, Crowe SF (2004) Persistence of cognitive effects after withdrawal from long-term benzodiazepine use: a meta-analysis. *Arch Clin Neuropsychol* 19: 437–454

Charlson F, Degenhardt L, McLaren J, Hall W, Lynskey M (2009) A systematic review of research examining benzodiazepine-related mortality. *Pharmacoepidemiol Drug Saf* 18: 93–103

Kripke DF, Langer RD, Kline LE (2012) Hypnotic association with mortality or cancer; a matched cohort study. *BMJ Open* 2: e000850

Lader M (2014) Benzodiazepine harm: how can it be reduced? *Br J Clin Pharmacol* 77: 295–301

Manthey L, van Loenen-Frösch F, Giltay EJ, van Veen T, Glashouwer K, Penninx BWJH, Zitman FG (2014) High dose benzodiazepines prolong reaction times in chronic users who have major depressive and/or anxiety disorders. *Br J Clin Pharmacol* 77: 571–577

Walton GR, Hayashi K, Bach P, Dong H, Kerr T, Ahamad K, Milloy MJ, Montaner J, Wood E (2016) The impact of benzodiazepine use on mortality among polysubstance users in Vancouver, Canada. *Public Health Rep* 131: 491-499

Cognitive-behavioral therapy:

Barlow DH, Gorman JM, Shear MK, Woods SW (2000) Cognitive-behavioral therapy, imipramine, or their combination for panic disorder: A randomized controlled trial. *JAMA* 283: 2529-2536

Bockting CL, Hollon SD, Jarrett RB, Kuyken W, Dobson K (2015) A lifetime approach to major depressive disorder: The contributions of psychological interventions in preventing relapse and recurrence. *Clin Psychol Rev* 41: 16-26

Durham RC, Chambers JA, Power KG, Sharp DM, Macdonald RR, Major KA, Dow MG, Gumley AI (2005) Long-term outcome of cognitive behaviour therapy clinical trials in central Scotland. *Health Technol Assess* 9: 1-174

Jarrett RB, Minhajuddin A, Vittengl JR, Clark LA, Thase ME (2016) Quantifying and qualifying the preventive effects of acute-phase cognitive therapy: Pathways to personalizing care. *J Consult Clin Psychol* 84: 365-376

Lelliott PT, Marks IM, Monteiro WO, Tsakiris F, Noshirvani H (1987) Agoraphobics 5 years after imipramine and exposure: Outcome and predictors. *J Nerv Ment Dis* 175: 599-605

Marks IM, Swinson RP, Basoglu M, Kuch K, Noshirvani H, O'Sullivan G, Lelliott PT, Kirby M, McNamee G, Sengun S *et al* (1993) Alprazolam and exposure alone and combined in panic disorder with agoraphobia. A controlled study in London and Toronto. *Br J Psychiat* 162: 776-778

Tolin DF (2010) Is cognitive-behavioral therapy more effective than other therapies? A meta-analytic review. *Clin Psychol Rev* 30: 710-720

Vittengl JR, Jarrett RB (2015) Cognitive therapy to prevent depressive relapse in adults. *Curr Opin Psychol* 4: 26–31

Effects on developing brain:

Bock N, Quentin DJ, Huther G, Moll GH, Banaschewski T, Rothenberger A (2005) Very early treatment with fluoxetine and reboxetine causing long-lasting change of the serotonin but not the noradrenaline transporter in the frontal cortex of rats. *World J Biol Psychiat* 6: 107–112

Bouet V, Klomp A, Freret T, Wylezinska-Arridge M, Lopez-Tremoleda J, Dauphin F, Boulouard M, Booij J, Gsell W, Reneman L (2012) Age-dependent effects of chronic fluoxetine treatment on the serotonergic system one week following treatment. *Psychopharmacology* 221: 329–339

Iñiguez SD, Warren BL, Bolanos-Guzman CA (2010) Short- and long-term functional consequences of fluoxetine exposure during adolescence in male rats *Biol Psychiat* 67: 1057–1066

Karanges E, Li KM, Motbey C, Callaghan PD, Katsifis A, McGregor IS (2011) Differential behavioural and neurochemical outcomes from chronic paroxetine treatment in adolescent and adult rats: a model of adverse antidepressant effects in human adolescents? *Int J Neuropsychopharmacol* 14: 491–504

Shrestha SS, Nelson EE, Liow JS, Gladding R, Lyoo CH, Noble PL, Morse C, Henter ID, Kruger J, Zhang B *et al* (2014) Fluoxetine administered to juvenile monkeys: effects on the serotonin transporter and behavior. *Am J Psychiat* 171: 323-331

Wegerer V, Moll GH, Bagli M, Rothenberger A, Ruther E, Huether G (1999) Persistently increased density of serotonin transporters in the frontal cortex of rats treated with fluoxetine during early juvenile life. *J Child Adolesc Psychopharmacol* 9: 13–24

Whittington CJ, Kendall T, Fonagy P, Cottrell D, Cotgrove A, Boddington E (2004) Selective serotonin reuptake inhibitors in childhood depression: systematic review of published versus unpublished data. *Lancet* 363: 1341–1345

Wohlfarth TD, van Zwieten BJ, Lekkerkerker FJ, Gispen-de Wied CC, Ruis JR, Elferink AJ, Storosum JG (2006) Antidepressants use in children and adolescents and the risk of suicide. *Eur Neuropsychopharmac*ol 16: 79–83

General clinical research and validity of mental disorder constructs

Ioannidis JPA (2005) Why most published research findings are false. *PLOS Med* 2: e124

Ioannidis JPA (2016) Why most clinical research is not useful. *PLOS Med* 13: e1002049

Margraf J (2015) Magritte’s mystery and DSM’s diagnoses. *Observer* 28, March 2015

McNally RJ, Robinaugh DJ, Wu GWY, Wang L, Deserno M, Borsboom D (2015) Mental disorders as causal systems: a network approach to posttraumatic stress disorder. *Clin Psychol Sci* 3: 836–849

Psychosis and dopamine:

Aderhold V, Weinmann S, Hägele C, Heinz A (2015) Frontal brain volume reduction due to antipsychotic drugs? *Nervenarzt* 86: 302-323

Chouinard G, Jones BD, Annable L (1978) Neuroleptic-induced supersensitivity psychosis. *Am J Psychiat* 135: 1409–1410

Chouinard G, Chouinard VA (2008) Atypical antipsychotics. CATIE study, drug-induced movement disorder and resulting iatrogenic psychiatric-like symptoms, supersensitivity rebound psychosis and withdrawal discontinuation syndromes. *Psychother Psychosom* 77: 69–77

DeSisto MJ, Harding CM, McCormick RV, Ashikaga T, Brooks GW (1995) The Maine and Vermont three-decade studies of serious mental illness. I. Matched comparison of cross-sectional outcome. *Br J Psychiat* 167: 331-338

Gardos G, Cole JO (1976) Maintenance antipsychotic therapy: is the cure worse than the disease? *Am J Psychiat* 133: 32–36

Harrow M, Jobe TH (2007) Factors involved in outcome and recovery in schizophrenia patients not on antipsychotic medications: a 15-year multifollow-up study. *J Nerv Ment Dis* 195: 406-414

Harrow M, Jobe TH, Faull RN (2012) Do all schizophrenia patients need antipsychotic treatment continuously throughout their lifetime? A 20-year longitudinal study. *Psychol Med* 17: 1–11

Harrow M, Jobe TH, Faull RN (2014) Does treatment of schizophrenia with antipsychotic medications eliminate or reduce psychosis? A 20-year multi-follow-up study. *Psychol Med* 44: 3007-16

Ho BC Andreasen NC Ziebell S (2011) Long-term antipsychotic treatment and brain volumes: a longitudinal study of first-episode schizophrenia. *Arch Gen Psychiat* 68: 128-137

Li M (2016) Antipsychotic-induced sensitization and tolerance: Behavioral characteristics, developmental impacts, and neurobiological mechanisms. *J Psychopharmacol* 30: 749-770

Moncrieff J (2015) Antipsychotic maintenance treatment: time to rethink? *PLOS Med* 12: e1001861

Moncrieff J, Leo J (2010) A systematic review of the effects of antipsychotic drugs on brain volume. *Psychol Med* 20: 1–14

Oda Y, Kanahara N, Iyo M (2015) Alterations of dopamine D2 receptors and related receptor-interacting proteins in schizophrenia: the pivotal position of dopamine supersensitivity psychosis in treatment-resistant schizophrenia. *Int J Mol Sci* 16: 30144–30163

Samaha A, Seeman P, Stewart J, Rajabi H, Kapur S (2007) Breakthrough dopamine supersensitivity during ongoing antipsychotic treatment leads to treatment failure over time. *J Neurosci* 27: 2979-2986

Seeman P, Weinshenker D, Quirion R, Srivastava LK, Bhardwaj SK, Grandy DK, Premont RT, Sotnikova TD, Boksa P, El-Ghundi M *et al* (2005) Dopamine supersensitivity correlates with D2High states, implying many paths to psychosis. *Proc Nat Acad Sci USA* 102: 3513-3518

Sohler N, Adams BG, Barnes DM, Cohen GH, Prins SJ, Schwartz S (2015) Weighing the evidence for harm from long-term treatment with antipsychotic medications: A systematic review. *Am J Orthopsychiatry* doi: 10.1037/ort0000106

Torres US, Duran FL, Schaufelberger MS, Crippa JA, Louzã MR, Sallet PC, Kanegusuku CY, Elkis H, Gattaz WF, Bassitt DP *et al* (2016) Patterns of regional gray matter loss at different stages of schizophrenia: A multisite, cross-sectional VBM study in first-episode and chronic illness. *Neuroimage Clin* 12: 1-15

Wunderink L, Nieboer RM, Wiersma D, Sytema S, Nienhuis FJ (2013) Recovery in remitted first-episode psychosis at 7 years of follow-up of an early dose reduction/discontinuation or maintenance treatment strategy: long-term follow-up of a 2-year randomized clinical trial. *JAMA Psychiatry* 70: 913-920
